# Environmental Surveillance through Next-Generation Sequencing to Unveil the Diversity of Human Enteroviruses beyond the Reported Clinical Cases

**DOI:** 10.3390/v13010120

**Published:** 2021-01-17

**Authors:** Andrés Lizasoain, Daiana Mir, Gisella Masachessi, Adrián Farías, Nélida Rodríguez-Osorio, Matías Victoria, Silvia Nates, Rodney Colina

**Affiliations:** 1 Laboratorio de Virología Molecular, Departamento de Ciencias Biológicas, Centro Universitario Regional del Litoral Norte, Universidad de la República, Salto 50000, Uruguay; andres.lizasoain.cuelho@gmail.com (A.L.); matvicmon@yahoo.com (M.V.); 2 Unidad de Genómica y Bioinformática, Departamento de Ciencias Biológicas, Centro Universitario Regional del Litoral Norte, Universidad de la República, Salto 50000, Uruguay; dmir@unorte.edu.uy (D.M.); nelida.rodriguez@unorte.edu.uy (N.R.-O.); 3 Laboratorio de Gastroenteritis Virales y Sarampión, Instituto de Virología Dr. J. M. Vanella, Facultad de Ciencias Médicas, Universidad Nacional de Córdoba, Córdoba 5000, Argentina; gmasachessi@fcm.unc.edu.ar (G.M.); adrianalefarias@hotmail.com (A.F.); snates@fcm.unc.edu.ar (S.N.)

**Keywords:** enterovirus, poliovirus, echovirus, coxsackievirus, surveillance, wastewater-based epidemiology, aseptic meningitis, outbreak, phylogenetic analysis, next-generation sequencing

## Abstract

The knowledge about circulation of Human Enteroviruses (EVs) obtained through medical diagnosis in Argentina is scarce. Wastewater samples monthly collected in Córdoba, Argentina during 2011–2012, and then in 2017–2018 were retrospectively studied to assess the diversity of EVs in the community. Partial VP1 gene was amplified by PCR from wastewater concentrates, and amplicons were subject of next-generation sequencing and genetic analyses. There were 41 EVs detected, from which ~50% had not been previously reported in Argentina. Most of the characterized EVs (60%) were detected at both sampling periods, with similar values of intratype nucleotide diversity. Exceptions were enterovirus A71, coxsackievirus B4, echovirus 14, and echovirus 30, which diversified in 2017–2018. There was a predominance of types from EV-C in 2017–2018, evidencing a common circulation of these types throughout the year in the community. Interestingly, high genetic similarity was evidenced among environmental strains of echovirus 30 circulating in 2011–2012 and co-temporal isolates obtained from patients suffering aseptic meningitis in different locations of Argentina. This study provides an updated insight about EVs circulating in an important region of South America, and suggests a valuable role of wastewater-based epidemiology in predicting outbreaks before the onset of cases in the community.

## 1. Introduction

Human Enteroviruses (EVs) comprise several viral types belonging to classical groups’ coxsackievirus (CV) A and B, echovirus (E), numbered enterovirus (EV) and poliovirus (PV). Additionally, EVs are classified into four viral species (EV-A-EV-D) inside the genus *Enterovirus* of the family *Picornaviridae* [1].

Frequently, infections are mild or asymptomatic [2], but a small fraction of them lead to diseases such as aseptic meningitis (AM), encephalitis, acute hemorrhagic conjunctivitis (AHC), hand-foot-and-mouth disease (HFMD), or acute flaccid paralysis (AFP), among others [3,4]. The primary mode of EVs transmission remains the fecal-oral route. However, respiratory aerosols, contact with objects or surfaces contaminated with virions, as well as consumption of contaminated water also raise the risk of infection [5,6]. Infections by EVs are ubiquitous among populations; and since the primary replication site for these viruses is the digestive tract, large amounts of viral particles are excreted in feces during either symptomatic or asymptomatic infections [7]. Consequently, when suitable methods are employed, the study of wastewater samples allows unveiling of the diversity of EVs in the population that surrounds the sampling points [8,9].

South America has been identified as being among the regions of the world with less available information about the genetic diversity of its circulating EVs strains [10]. In particular, Argentina has extensively studied the molecular epidemiology of echovirus 30, a member of EV-B related with AM [11,12,13]. Additionally, there are some reports regarding outbreaks of different diseases caused by EVs [14,15,16,17]. Previous wastewater-based epidemiology studies have provided valuable information about EVs types circulating in Argentina [18,19,20,21], thus complementing clinical analysis of samples from patients with diseases associated with EVs reported in the 1990s and early 2000s [22,23]. Most of these environmental studies were carried out in the Province of Córdoba, but they were biased towards poliovirus isolation and viral strains characterization [19] or were based on Sanger sequencing of a PCR product obtained directly from the wastewater specimens [20,21]. Considering that wastewater is a complex sample in which there normally occurs a mixture of several EVs types as a result of multiple excretion events in the population [24,25], and due to the inherent diversity of types when EVs are assessed, these approaches had limitations for providing a wide understanding on the diversity of circulating EVs. Beyond all efforts for characterizing EVs’ presence in Argentina, the knowledge about the circulation of more than 100 types of these viruses in the country is scarce.

In recent years, different studies have employed next-generation sequencing techniques to describe diversity, seasonality, and/or to find particular emerging types of EVs in environmental samples, achieving an in-depth view of the real diversity enclosed by each sample [25,26,27,28].

The present wastewater-based epidemiology study constitutes a great contribution to the knowledge about the circulation of EVs in Argentina and provides evidence about the usefulness of the applied methodological approach for anticipating possible outbreaks.

## 2. Materials and Methods

### 2.1. Samples

Viral concentrates (15 mL)–which were obtained as previously described [29] by a precipitation method with polyethylene glycol (PEG) from wastewater samples (1500 mL each)–were retrospectively studied. Samples were monthly collected from the inlet of Bajo Grande wastewater treatment plant (WTP) from Córdoba City in two periods; the first period took place from January 2011 until December 2012, and the second period took place from March 2017 until February 2018. Córdoba city is the capital of the province of Córdoba, located in the central region of Argentina, and is the second most populated city of the country with approximately 1,329,604 inhabitants and a population density of 5,978 people per square mile [30]. The Bajo Grande WTP represents the sewage discharges from about 61% of the population and this facility does not treat industrial wastewater.

### 2.2. Human Enteroviruses Detection and Sequencing

Viral genomes were extracted from 140 µL of viral concentrate using the QIAamp® Viral RNA Mini Kit (Qiagen™, Hilden, Germany) according to manufacturer’s instructions. A RT-nested PCR method [31] was used to amplify a partial segment of VP1-coding segment (2602–2977 positions in the genome of PV1 Mahoney strain. GenBank accession number J02281), with primers AN32-AN35 in the reverse transcription (Revert Aid RT Thermo Scientific™, Carlsbad, CA, USA) and primers 222/224 (with Ranger DNA Polymerase Master Mix, Meridian Bioscience™, Cincinnati, OH, USA) and AN88/AN89 (with Platinum™ SuperFi™ Taq DNA Polymerase, Invitrogen™, Carlsbad, CA, USA), in first and second PCR rounds, respectively. At the second round, AN88 and AN89 primers [31] were modified by addition of Illumina Universal Adapter sequences at the 5´ends, according to the protocol of library preparation for metagenomic sequencing in Illumina MiSeq platform (Illumina Inc., San Diego, CA, USA) [32]. Macrogen Inc. Next Generation Sequencing Service (Seoul, Republic of Korea) prepared sequencing libraries. Samples were sequenced on Illumina MiSeq 2 × 300 bp, producing paired end reads. Libraries from 2011–2012 and 2017–2018 were sequenced in two independent runs.

### 2.3. Bioinformatics Pipeline

Raw Illumina reads were paired using *merge pairs* algorithm with a minimum overlap length of 50 bp in VSEARCH v2.11 [33]. The resulting contigs were filtered out if their lengths were <100 bp, and if they contained homopolymer tracks >8 bp in length. Quality trimming of reads was performed by using --*fastq_filter* command and contigs with more than 1.0 total expected errors (--*fastq_maxee* 1.0) were discarded. In order to save time in further analyses, a dereplication step was performed using VSEARCH --*derep_fulllength* algorithm on the processed contigs to find unique sequences by clustering at 100% sequence identity. Singletons (sequences that appear only once in the sample) and chimeras (abskew = 2) were removed.

Clusters of contigs were generated with VSEARCH using identity criterion of 97%, adopting a representative sequence (centroid sequence) of each cluster for further analyses. Finally, centroids sequences were mapped by VSEARCH’s *--usearch_global* at 80% sequence identity against a Customized Human Enteroviruses Database (CHED), composed of 30,850 sequences of the viral capsid VP1 protein-coding region downloaded from NCBI nucleotide database (CHED available upon requested).

### 2.4. Phylogenetic Analyses and Genetic Diversity

A subset from CHED was generated by removing closely related sequences within each EV type. To do this, sequences were grouped by similarity (97%) with the CD-HIT-EST tool [34] and only one sequence per cluster was selected.

An alignment for each EV species was constructed including the CHED representative sequences and centroid sequences from this study. A maximum likelihood (ML) phylogenetic tree was inferred for the completed data set of each EV species by using FastTree v2.1 software [35] with the GTR nucleotide substitution model. Lengths were rescaled to optimize the Gamma20 likelihood after using the CAT approximation to optimize the trees. The reliability of the phylogenies was estimated with the approximate likelihood-ratio test (aLRT) based on a Shimodaira–Hasegawa-like procedure [36,37]. Phylogenetic trees were visualized with FigTree v1.4.3.

Additionally, nucleotide identities values were calculated in BioEdit software v7.2.5 [38] for each pair of centroid sequences exclusively for each one of the types detected in both periods (2011–2012 and 2017–2018).

### 2.5. Backgrounds of Human Enteroviruses Detection in Argentina. Link between Environmental and Clinical Isolates

A bibliographic and nucleotide database search was conducted in order to know the most recent report in Argentina—either for environmental or clinical isolates—of each EV detected in this study.

Additionally, nucleotide sequences of clinical isolates—which were obtained from AM cases reported in different locations of Argentina between 1998 and 2012 [13]—were retrieved from GenBank nucleotide database for a genetic comparison with strains detected in wastewater by this study. Comparisons were performed through a matrix of nucleotide identity constructed in BioEdit software v7.2.5 [38]. GenBank accession numbers and information regarding location and year of detection for clinical isolates used in this analysis are available in Table S1.

## 3. Results

### 3.1. Taxonomic Allocation and Distribution of Human Enteroviruses Species’ Frequency and Abundance

Number of merged reads (contigs) per sample ranged between 63,293 and 118,027 (average 106,328 ± 11,680) for 2011–2012 and between 132,716 and 300,971 (average 179,030 ± 46,968) for 2017–2018. The average loss of contigs after the two-filter process for 2011–2012 and 2017–2018 was 14% ± 4.1% and 15% ± 5.3%, respectively.

When considering all samples, it was observed that more than 97% of the total filtered contigs mapped to EVs sequences. At the 2011–2012 and the 2017–2018 sampling periods, on average 7 and 11 different EVs types per sample were detected, respectively (Table 1).

**Figure 1:**
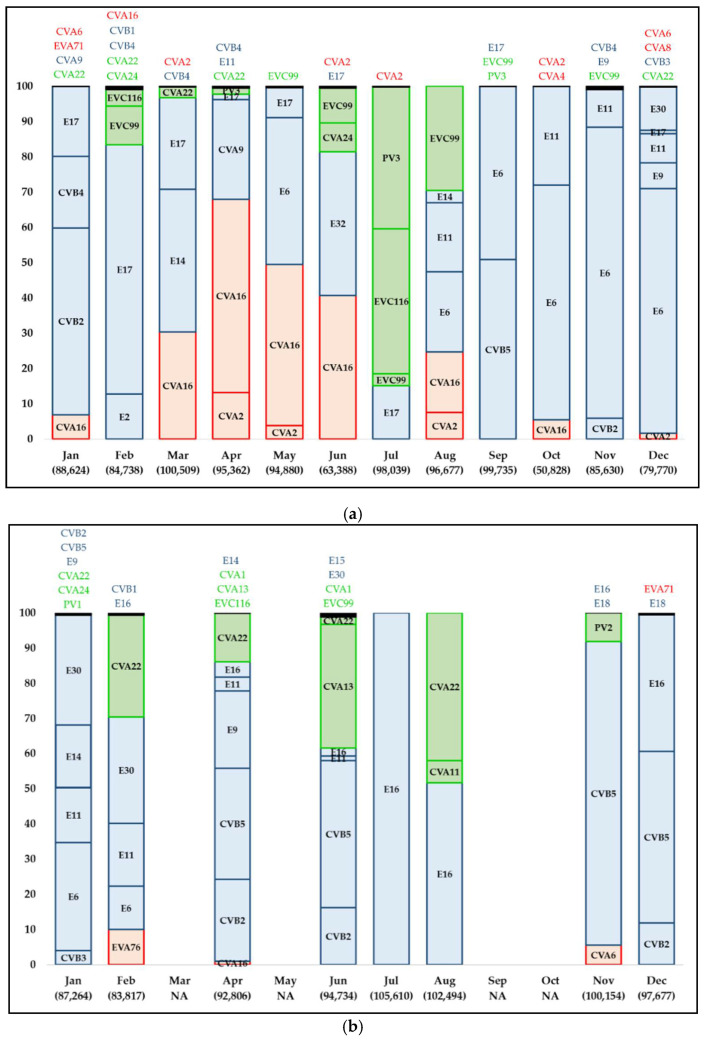

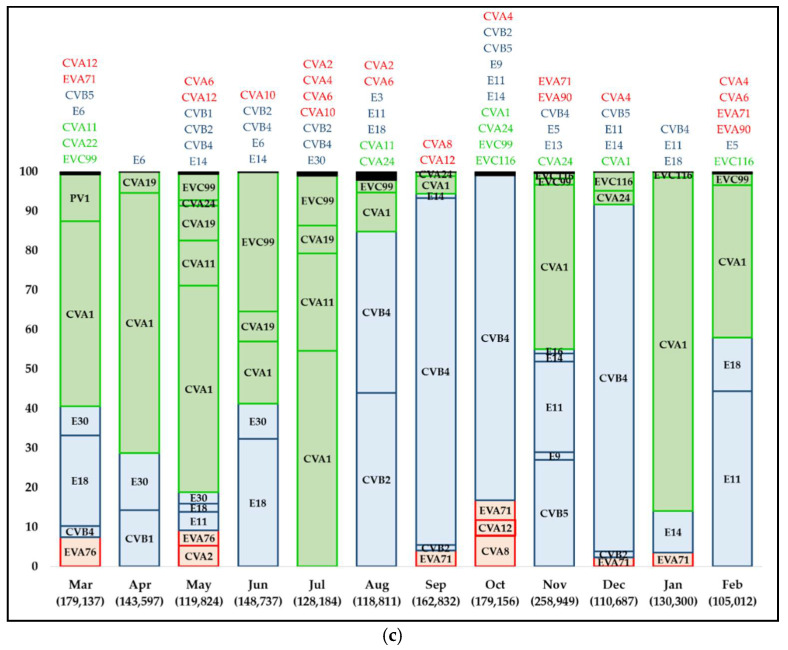
Abundance of contigs mapping to different Human Enteroviruses types/species in wastewater samples monthly collected in Córdoba City (Argentina) during 2011 (**a**), 2012 (**b**), and 2017–2018 (**c**). The bar-graphs show the relative abundance in percentage of contigs mapping to different Human Enteroviruses types for each sample. Types represented by less than 1% of the mapped contigs, abundances which are accumulated in a black portion, appear named and colored according the species at the top of each bar. Types belonging to species A are in red, to species B in blue, and to species C in green. Samples from March, May, September, and October 2012 were not assayed (NA) due to low volumes of available viral concentrates. The total number of contigs mapping to Human Enteroviruses is indicated between parentheses for each sample. CVA: coxsackievirus type A; CVB: coxsackievirus type B; E: echovirus; EVA: enterovirus type A; EVB: enterovirus type B; EVC: enterovirus type C; PV: poliovirus.

Throughout both sampling periods, EV-B was the most frequent and abundant detected species, while EV-C was detected in all the samples (like EV-B) during 2017–2018, with increased abundance compared to 2011–2012. EV-A was detected in a low abundance throughout the entire sampling period; with the exception of March through June 2011, when EV-A abundance was relatively higher compared to the one from the remaining months of the sampling (Figure 1).

### 3.2. Human Enteroviruses Characterization

In total, 41 different EVs types were detected: 10 types belonging to EV-A, 20 types to EV-B and 11 types to EV-C. Twenty-four out of 41 types were detected in both sampling periods (Figure 1; Figure S1).

According to the period of detection (2011–2012 and 2017–2018), several phylogenetic clusters were characterized for most of the different types (Figure S1). The majority of the phylogenetic clusters were detected exclusively during one of the sampling periods, but a lower number of them were composed by highly related centroid sequences detected at both periods (black squares of Figure S1).

The dynamics of EVs types within the three detected species was quite heterogeneous throughout the sampling period (Figure 1). In 2011–2012 period (Figure 1a,b), the predominant types were CVA2, CVA16 (EV-A), CVB4, CVB5, E6, E11, E14, E16, E17, E30 (EV-B), CVA22, EVC99 (EV-C). In the period considered between 2017 and 2018 (Figure 1c) the prominent ones were EVA71, CVA4, CVA6, CVA12 (EV-A), CVB2, CVB4, E11, E14, E18, E30 (EV-B), CVA1, CVA19, CVA24, EVC99 (EV-C).

### 3.3. Nucleotide Identity

**Figure 2:**
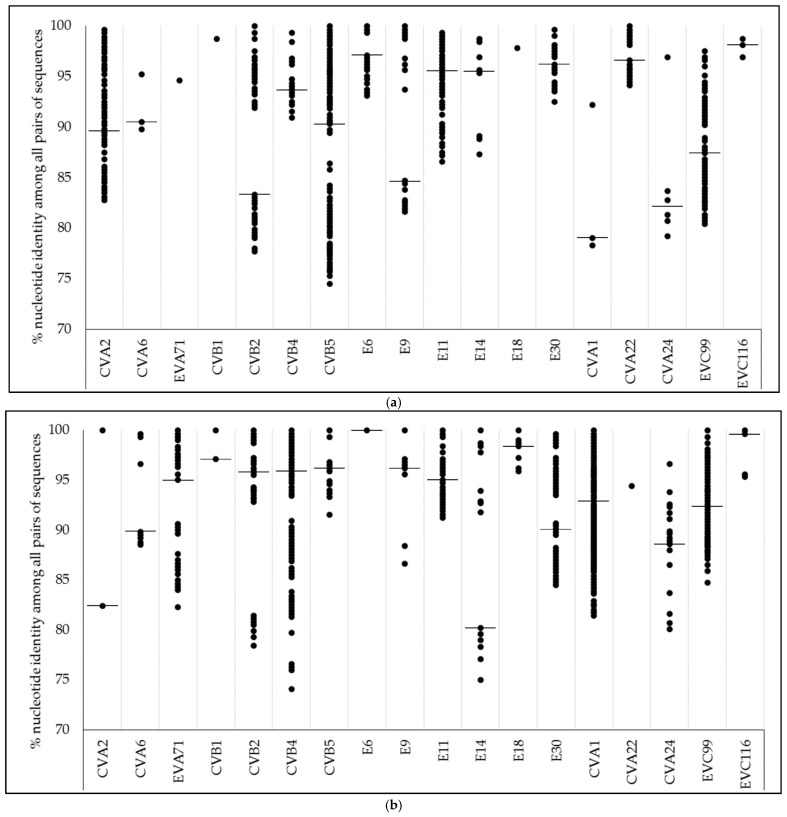
Nucleotide identity values over all the centroid sequences pairs for each Human Enterovirus type detected in wastewater samples from Córdoba City, Argentina in both periods (**a**) 2011–2012 and (**b**) 2017–2018. Horizontal bars indicate the median of nucleotide identity when more than a pair of centroid sequences was detected.

Values of nucleotide identity among all pairs of centroid sequences for EVs types detected at both sampling periods (2011–2012 and 2017–2018) were in the range of 74.1–100%, when all types were considered (Figure 2). The range of genetic identity values was similar for both periods, with few exceptions: wider ranges in 2017–2018 than in 2011–2012 were observed for EVA71, CVB4, E14, and E30; meanwhile the range of identity for CVB5 decreased in 2017–2018 compared with 2011–2012.

### 3.4. Backgrounds of Human Enteroviruses Detection in Argentina

After the bibliographic and nucleotide database search, we noted that this study constitutes the first detection in Argentina for 40% (15/38) of the non-polio EVs types detected here, mostly EV-A and EV-C types. Additionally, 17% (4/23) of non-polio EV types (CVA2, EVA71, CVB1, and E11) with previous evidence of circulation in the country are types whose detection is barely mentioned in previous studies and are without a register in GenBank. The majority of EV-B types were previously detected in Argentina (17/19), although last reports were from 15 years ago (or more) for ~50% of them (Table 2).

### 3.5. Link between Human Enteroviruses Detected in Wastewater and Clinical Isolates

Nucleotide identity percentages of centroid sequences of echovirus 30 from this study and those available in GenBank obtained during the study of sporadic cases and outbreaks of AM accounted during 1998–2012 in different locations of Argentina [13] were compared (Table 3).

Echovirus 30 strains detected in wastewater in 2011 and 2012 were closely related with clinical isolates reported during outbreaks of AM accounted in Buenos Aires Province and Chaco Province in 2011 and 2012 (95.9–99.6%), and with isolates reported from the study of two sporadic cases reported in 2012 in Córdoba (97.8–100%). Instead, strains detected in wastewater in 2017 were more related with strains of clinical isolates from the period from 1998 to 2008 (85.1–95.6%) than with those strains more recently detected from medical diagnosis of AM (83.3–88.2%) in Argentina.

## 4. Discussion

We have studied the genetic diversity of EVs presented in wastewater from Córdoba, Argentina along two different sampling periods separated by a span of 5 years. Our analysis supports the circulation of 41 different EVs types during both periods, of which 38 of them are non-polio EVs.

The average numbers of types detected per 100,000 contigs were identical in the periods of 2011–2012 and 2017–2018 (Table 1), suggesting that differences initially observed in the detected number of types among periods were caused by sequencing depth, instead of an increased circulation of EV types during 2017–2018. To the best of our knowledge, this study represents the first detection in Argentina of 15 of these 38 EV types, being in the majority first detections of EV-A (6/15) and EV-C (6/15) types in the country. Despite the fact that most of the EV-B types detected in this study had been previously reported in Argentina, half of them, the last record in the country was at least 15 years ago (Table 2). Therefore, the results of our work contribute to a better understanding and updating of the epidemiology and circulation dynamics of EV in this South American country.

This study documented for Córdoba, Argentina, the recent circulation of several well-known EVs types, to be linked to different diseases such as HFMD (coxsackievirus A6, A10, A16), AM (echovirus 6, 30, coxsackievirus A9, B4), AHC (coxsackievirus A24), AFP (echovirus 14 and enterovirus A71), among others [4]. Such diversity of EVs linked to different diseases deserves attention from the public health point of view since when it is interpreted together with the scarce data obtained from medical diagnoses, this suggests, in the worst possible scenario, an under-reporting of several diseases with important implications for the population´s health. Additionally, environmental samples frequently contain viable EVs [29,41,42,43] which means that several of the detected EVs types represented certain risk of infection to those who were exposed to wastewater or to other polluted environmental matrices.

Different EVs types present different circulation and/or predominance patterns along time [44]. The five years gap between both assayed periods limited our study in addressing the epidemiologic patterns followed by each EV type since there was a loss of information during this impasse. Nonetheless, some results about EVs types’ diversity along the sampling of this study deserve highlighting. First, 60% (24/41) of the types detected in our study circulated both during 2011–2012 and during 2017–2018 (Figure S1), which could be either due to a prolonged circulation given by an endemic behavior, or resulted from recurrent entries into Córdoba that coincided with our sampling periods. Second, EVs types circulated under similar values of genetic diversity at each period (Figure 2). Nevertheless, there was a considerable expansion of the range of nucleotide identity towards lower values for EVA71 (EV-A), CVB4, E14, and E30 (EV-B) sequences in 2017–2018, which probably was a consequence of the introduction of phylogenetically distant strains at intra-type level after 2011–2012 (Figure S1). This highlights the dynamic circulation and indicates the need for future detailed inspection of clinical cases, potentially linked with these EV types in the community. Third, for some consecutive months (i.e., June–July 2012, November–December 2017) the diversity of EVs types abruptly changed (Figure 1). Other studies conducted in different parts of the world have also documented this complete (or almost complete) exchange of types from one month to another [27,45]. Diverse factors such as the differential seasonal fluctuations of types, immunity generation into population, occurrence of large outbreaks of different diseases, or the introduction of new viral variants, among many others, could act as driving forces of this pattern. Unfortunately, most of the environmental studies of EVs which used next-generation sequencing have been conducted during short sampling campaigns as in our case [26,27,28,45,46,47]. Long-term environmental surveillance projects could be useful to elucidate epidemiological patterns for EVs beyond the bounded subset of EVs commonly detected when clinical specimens obtained from AM or AFP cases are studied.

Another interesting aspect of our study that deserves attention is the variation in diversity of EV species along the sampling period (Figure 1). The most evident change happened in the abundance of EV-C types during 2017–2018 in comparison with 2011–2012. This change was mainly driven by the ignition of CVA1 during 2017–2018, although other types such as CVA11 or CVA19 also contributed to it. In line with the herein achieved results, recent studies have documented high percentages of detection of EV-C types in environmental samples from distant points of the world [26,48,49]. Besides many coxsackievirus-A types and some numbered enteroviruses, this species includes three poliovirus types [50]. During many years, global efforts against EVs transmission were focused on the eradication of wild polioviruses through vaccination [51]. Previous studies have speculated about a “vacated niche” once poliovirus is eradicated, and based on the close genetic relation with poliovirus and the plasticity of their genomes, coxsackieviruses-A types from EV-C have been proposed as a potential successor of poliovirus in a poliovirus-free world [52]. Nevertheless, a recent study [53] did not find an increased circulation of EV-C types in a period characterized by less poliovirus circulation in a community, although this absence of detection of EV-C types could be the result of a bias introduced by the employed methods [54]. Therefore, the emergence of EV-C types as endemic viruses due to less poliovirus circulating in humans remains speculative. Nonetheless, the relevance of their silent spreading into a community should be the subject of future studies oriented towards a better understanding of the behavior of these rarely reported viruses from diagnoses [45,48,55].

Despite Argentina immunizing children with the live-attenuated Oral Poliovirus Vaccine (OPV) during the sampling periods of our study, we detected PV in 19% (6/32) of the collected samples. This low frequency of detection could probably be due to an amount of OPV excretors lower than the amount of other non-polio enteroviruses excretors in the population, with the consequence of PV strains´ dilution in the mixture of diverse EV types when wastewater samples are assayed with primers designed for broad target specificity. Nevertheless, other issues such as low catchment of immunized children shedding PV into the wastewater system, or different recovery efficiencies for different enteroviruses during the wastewater concentration procedure could be some of the factors affecting the PV detection rate in our study.

Unfortunately, there are few studies reporting EVs from medical diagnosis in Argentina [12,13,17,22,23] and data encompassing our sampling are not enough to make a detailed comparison among clinical and environmental strains for the 41 types herein detected. Nevertheless, echovirus 30 has probably been the most extensively studied EV type in Argentina [11,12,13], and there are several nucleotide sequences publicly available. This allowed us to conduct a retrospective comparison of echovirus 30 strains detected in this study with isolates obtained during outbreaks and sporadic cases of AM accounted in Córdoba and in other provinces of Argentina [13]. This comparison evidenced a close phylogenetic relation among environmental strains detected in Córdoba in samples taken from 2011–2012, and clinical isolates both from Córdoba and other locations from Argentina reported during those same years. Interestingly, our results show that when sporadic cases of AM linked with echovirus 30 were reported in Córdoba (March 2012) the virus had been circulating in the wastewater of the city for at least three months prior (Table 3). Similarly, a previous study [56] reported the silent circulation of echovirus 30 in the environment before the onset of a large outbreak of AM accounted in 2009 in Finland. However, a study conducted in Russia [57], failed in associating clinical and environmental isolates of echovirus 30 in the same temporal and geographical scenario, reporting echovirus 30 almost exclusively from medical diagnosis of AM. This emphasizes the use of highly sensitive and effective methods to unveil the complex diversity of EVs in environmental samples when wastewater-based epidemiology approaches are conducted. The lack of reports of AM linked to echovirus 30 in Argentina since 2012 made it impossible for us to compare the characterized environmental strains with contemporaneous clinical isolates. A link between environmental and clinical isolates of different EVs obtained in the same temporal and geographical range has been extensively documented [57,58,59]. Nevertheless, our study suggests that echovirus 30 strains detected in the environment of a specific location could predict future epidemics beyond local boundaries. This has a valuable application in a potential national environmental surveillance system based on sentinel points for anticipating the occurrence of outbreaks.

Despite the fact that our approach is an improvement compared to the classic diagnostic methods routinely used across laboratories [60], and the fact that it allowed the detection of up to 16 different EVs per sample; other similar approaches have shown a higher sensitivity [26,45]. Different issues such as the wastewater concentration method, the amount of template used in RNA extraction, RT-PCR methods, or some details regarding the sampling strategy or, indeed, epidemiological aspects could be affecting the number of types detected per sample among different studies. Future comparative studies are needed in order to know the advantages and disadvantages of each approach to evaluate their applicability in accomplishing different specific goals.

In summary, we implemented a wastewater-based epidemiology approach with next-generation sequencing of a viral gene amplified by PCR in order to depict the diversity of EVs circulating in Córdoba, Argentina. This leads to a better characterization of the presence of these viruses in the country, showing how useful this kind of approach is—when continuously conducted from the wastewater of a community—to know in advance the circulation of EVs potentially hazardous for the public health.

## Figures and Tables

**Table 1:** Variation in the abundance and efficiency of the typing process for contigs obtained by next-generation sequencing of Human Enteroviruses detected in wastewater samples.

Sample	No. Contigs *	No. Contigs Represented by Centroids (*n*) after Clusterization, and Quality Filtering	No. Contigs and Centroids (*n*) Mapping to CHED §	% of Mapped Contigs	No. Types Detected	Normalization (No. Types Detected/100,000 Contigs)
Jan-11	100,139	88,677 (15)	88,624 (14)	99.940	8	9
Feb-11	94,803	84,740 (12)	84,738 (11)	99.998	9	11
Mar-11	113,365	100,538 (11)	100,509 (8)	99.971	6	6
Apr-11	109,599	95,364 (13)	95,362 (12)	99.998	8	8
May-11	112,312	94,882 (12)	94,880 (11)	99.998	5	5
Jun-11	108,516	94,081 (10)	63,388 (9)	67.376	6	9
Jul-11	109,250	98,124 (11)	98,039 (5)	99.913	5	5
Aug-11	110,859	96,682 (11)	96,677 (10)	99.995	6	6
Sep-11	110,270	99,735 (5)	99,735 (5)	100	5	5
Oct-11	63,293	50,828 (7)	50,828 (7)	100	5	10
Nov-11	110,198	85,630 (11)	85,630 (11)	100	6	7
Dec-11	101,611	79,770 (14)	79,770 (14)	100	10	13
Jan-12	103,427	87,296 (18)	87,264 (17)	99.963	11	13
Feb-12	100,802	83,832 (13)	83,817 (11)	99.982	7	8
Apr-12	107,125	92,808 (17)	92,806 (16)	99.998	11	12
Jun-12	110,616	94,899 (22)	94,734 (18)	99.826	10	11
Jul-12	114,277	105,637 (7)	105,610 (1)	99.974	1	1
Aug-12	111,862	102,504 (6)	102,494 (3)	99.990	3	3
Nov-12	116,199	100,154 (11)	100,154 (11)	100	5	5
Dec-12	118,027	97,677 (12)	97,677 (12)	100	5	5
**2011–2012**	**106,328** **(±11,680)**	**91,693 (12)** **loss of 14% ± 4.1%**	**90,137 (10)**	**98.346**	**7**	**8**
Mar-17	204,856	179,282 (37)	179,137 (35)	99.919	13	7
Apr-17	205,429	187,007 (29)	143,597 (21)	76.787	5	3
May-17	160,517	119,872 (79)	119,824 (76)	99.960	16	13
Jun-17	175,947	150,860 (33)	148,737 (25)	98.593	10	7
Jul-17	155,278	128,309 (44)	128,184 (42)	99.903	11	9
Aug-17	133,853	118,825 (25)	118,811 (22)	99.988	11	9
Sep-17	179,041	165,549 (19)	162,832 (11)	98.359	8	5
Oct-17	205,001	179,162 (27)	179,156 (26)	99.997	14	8
Nov-17	300,971	259,183 (54)	258,949 (49)	99.910	14	5
Dec-17	132,716	111,404 (24)	110,687 (20)	99.356	10	9
Jan-18	158,591	130,304 (33)	130,300 (31)	99.997	7	5
Feb-18	136,157	105,016 (28)	105,012 (27)	99.996	10	10
**2017–2018**	**179,030** **(± 46,968)**	**152,898 (36)** **loss of 15% ± 5.3%**	**148,769 (32)**	**97.730**	**11**	**8**

* Resulting from raw paired reads merging. § CHED: Customized Human Enteroviruses Database (centroids were mapped against it for typing). Average values for 2011–2012 and 2017–2018 are in boldface.

**Table 2:** Backgrounds of circulation in Argentina for Human Enteroviruses types detected in this study.

Type	More Recent Reported Isolates	Year/Period	Location	Source	Accession Number	Reference
CVA2	Just mentioned ^a^	1991–1998	Unknown	AM	Unavailable	[23]
CVA4	Unknown	---	---	---	---	---
CVA6	CVA6/BSAS/Arg001/2018	2018	Buenos Aires	HFMD	MK867799	[39]
CVA8	Unknown	---	---	---	---	---
CVA10	Unknown	---	---	---	---	---
CVA12	Unknown	---	---	---	---	---
CVA16	707F99	Unknown	Unknown	Unknown	AF290092	[11]
EVA71	Just mentioned ^a^	1991–1998	Unknown	AM & AFP	Unavailable	[23]
EVA76	Unknown	---	---	---	---	---
EVA90	Unknown	---	---	---	---	---
CVA9	Cba_Mar_2014_2	2014	Córdoba	Wastewater	MK435332	[21]
CVB1	Just mentioned ^a^	1991–1998	Unknown	AFP	Unavailable	[23]
CVB2	AR1530-3-03	2003	Buenos Aires	Neurological disease	DQ282630	[22]
CVB3	AR1450-49-02	2002	Buenos Aires	Neurological disease	DQ246722	[22]
CVB4	Cba_Mar_2010	2010	Córdoba	Wastewater	MK435329	[21]
CVB5	AR875-48-01	2001	Buenos Aires	Neurological disease	DQ246729	[22]
E2	AR1095-20-02	2002	Buenos Aires	Neurological disease	DQ282634	[22]
E3	Unknown	---	---	---	---	---
E5	AR1617-8-03	2003	Buenos Aires	Neurological disease	DQ282627	[22]
E6	Cba_Feb_2014_1	2014	Córdoba	Wastewater	MK435318	[21]
E9	AR1669_12_03	2003	Buenos Aires	Neurological disease	DQ246752	[22]
E11	Just mentioned ^a^	2005–2006	Buenos Aires	Surface water	Unavailable	[18]
E13	Cba_May_2013_1	2013	Córdoba	Wastewater	MK435334	[21]
E14	Cba_Set_2014	2014	Córdoba	Wastewater	MK435346	[21]
E15	Unknown	---	---	---	---	---
E16	Cba_Jun_2013	2013	Córdoba	Wastewater	MK435326	[21]
E17	875NE99	Unknown	Unknown	Unknown	AF290905	[11]
E18	AR2109-44-03	2003	Buenos Aires	Neurological disease	DQ246765	[22]
E30	CHA468-ARG12	2012	Chaco	AM	MK410196	[13]
E32	Unknown	---	---	---	---	---
CVA1	Unknown	---	---	---	---	---
CVA11	Unknown	---	---	---	---	---
CVA13	ARG98-10613/ARG98-10614	1998	Unknown	Unknown	DQ995636	[40]
CVA19	Unknown	---	---	---	---	---
CVA22	Unknown	---	---	---	---	---
CVA24	Cba_Aug_2013/Cba_Aug_2014	2013/2014	Córdoba	Wastewater	MK435313	[21]
EVC99	Unknown	---	---	---	---	---
EVC116	Unknown	---	---	---	---	---

ᵃ Human Enteroviruses (EVs) types mentioned as circulating in Argentina in an article, but without registry of nucleotide sequence in GenBank database. AM: Aseptic meningitis, AFP: acute flaccid paralysis, HFMD: Hand-foot-and-mouth disease.

**Table 3:** Nucleotide identities among echovirus 30 strains detected in wastewater from Córdoba (in 2011, 2012, and 2017) and clinical isolates obtained from outbreaks and sporadic cases of aseptic meningitis accounted in Argentina between 1998 and 2012 reported by Lema et al., 2019 [13].

	Aseptic Meningitis Outbreak Buenos Aires ᵃ (Oct 2011–Mar 2012)	Two Aseptic Meningitis Sporadic Cases Córdoba (Mar 2012)	Aseptic Meningitis Outbreak Chaco ᵃ (Feb 2012–Apr 2012)	Other Echovirus 30 Strains Detected in 1998–2008 in Argentina
E30/COR/ARG/Dec_2011	**95.9–99.3%**	**99%**	**97.8–99.3%**	87.9–92.2%
E30/COR/ARG/Jan_2012	**97.2–98.1%**	**97.80%**	**97.2–98.1%**	87.6–91.9%
E30/COR/ARG/Feb_2012	**97.5–98.4%**	**98.10%**	**97.5–98.4%**	87.3–92.2%
E30/COR/ARG/Feb_2012	**95.6–98.1%**	**97.80%**	**97.2–98.4%**	87.3–91.9%
E30/COR/ARG/Jun_2012	**96.2–99.6%**	**100%**	**98.1–99.6%**	87.9–92.2%
E30/COR/ARG/Mar_2017	86.4–87.3%	87.30%	86.1–87.3%	87.9–92.2%
E30/COR/ARG/Mar_2017	83.3–84.5%	84.50%	83.3–84.5%	85.1–91.0%
E30/COR/ARG/Mar_2017	84.2–85.8%	85.10%	84.5–85.8%	87.6–91.9%
E30/COR/ARG/Mar_2017	85.8–87.3%	86.70%	86.7–87.6%	86.4–95.6%
E30/COR/ARG/Mar_2017	84.5–86.7%	86.10%	85.4–86.7%	86.1–92.5%
E30/COR/ARG/Mar_2017	85.4–86.4%	86.40%	85.1–86.4%	87.0–93.5%
E30/COR/ARG/Apr_2017	85.4–87.0%	86.40%	86.4–87.3%	87.3–95.3%
E30/COR/ARG/May_2017	86.1–87.6%	87.00%	87.0–87.9%	86.7–95.3%
E30/COR/ARG/Jun_2017	85.8–87.3%	86.70%	86.7–87.6%	87.0–95.6%
E30/COR/ARG/Jul_2017	86.4–87.9%	87.30%	87.3–88.2%	87.6–95.3%

ᵃ Buenos Aires and Chaco Provinces are located 435 and 534 miles, respectively, from Province of Córdoba. Percentages in boldface show a close genetic relationship among environmental and clinical isolates circulating in Argentina in 2011–2012.

## Data Availability

Sequencing data is openly available in Sequence Read Archive (SRA) repository under BioProject number PRJNA689577.

## Supplementary Materials

The following are available online at https://www.mdpi.com/1999-4915/13/1/120/s1, Figure S1A–C: Maximum likelihood phylogenetic trees of Human Enterovirus species A (**A**), B (**B**), and C (**C**). Each tree was constructed with centroid sequences detected in wastewater samples from Córdoba City (Argentina) during this study and representative sequences of different Human Enteroviruses types reported elsewhere. Dotted lines projecting outward from branches represent centroid sequences. Colored circles and squares indicate the period(s) in which different phylogenetic clusters of this study were detected. aLRT-SH values at type-definition node levels were >0.7 for all types. The bar at the center of each phylogenetic tree denotes genetic distance. CVA: coxsackievirus type A; CVB: coxsackievirus type B; E: echovirus; EVA: enterovirus type A; EVB: enterovirus type B; EVC: enterovirus type C; PV: poliovirus. Table S1: Information about clinical isolates of echovirus 30 obtained from aseptic meningitis cases accounted in Argentina in 1998–2012 (reported by Lema et al. 2019 [13]), used in a comparison with echovirus 30 strains detected in wastewater by this study.
