# Supplementary material for: Environmental Surveillance through Next-Generation Sequencing to Unveil the Diversity of Human Enteroviruses beyond the Reported Clinical Cases
Source: Viruses. 2021 Jan 17;13(1):120. doi: 10.3390/v13010120 (PMC7829892; doi:10.3390/v13010120)
Supplement: Supplementary file 1 [file viruses-13-00120-s001.zip › Figure S1.pdf]

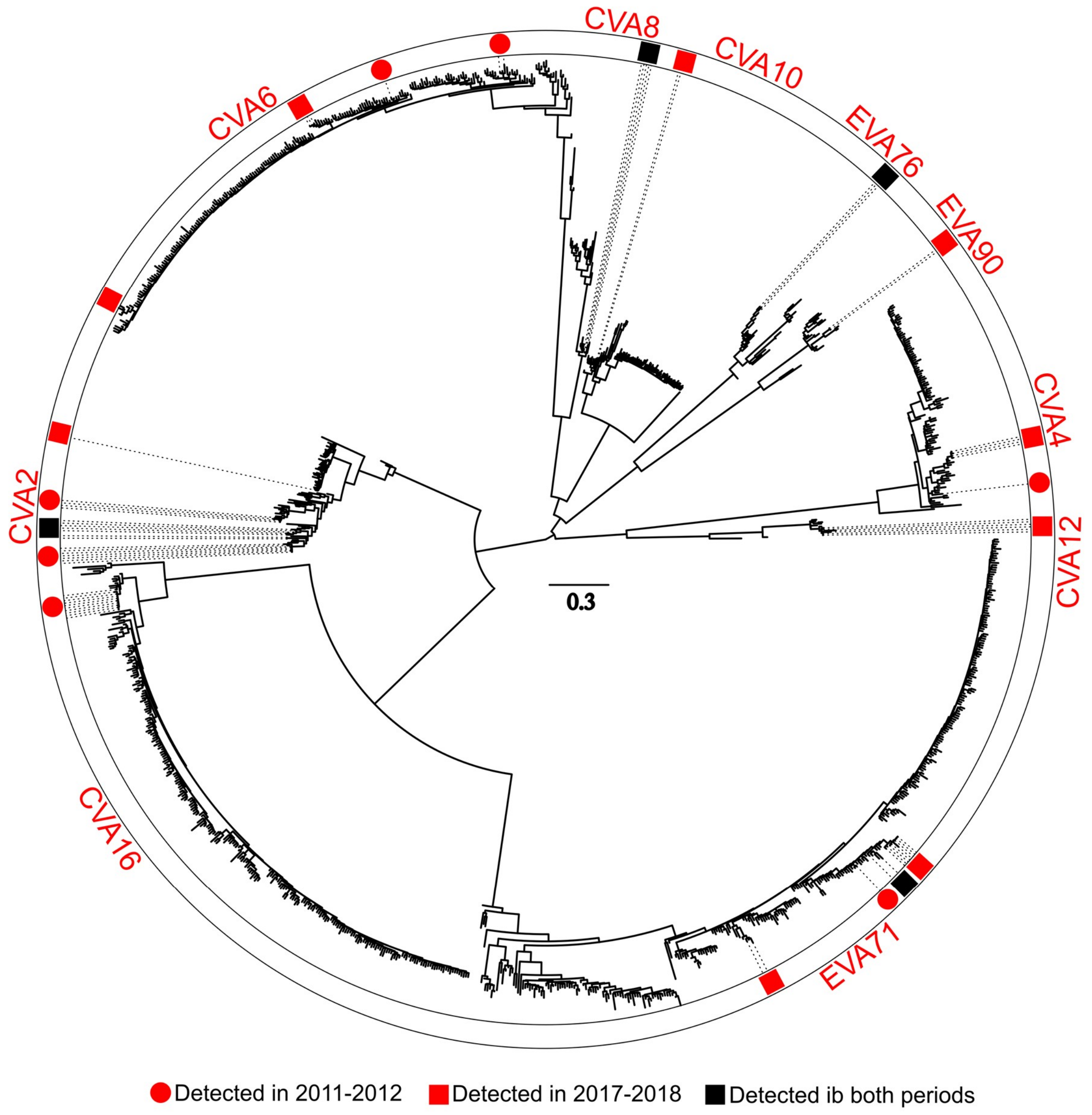
CVA8
CVA10
EVA76
EVA90
CVA4
CVA12
EVA71
CVA16
CVA2
CVA6
0.3
Detected in 2011-2012
Detected in 2017-2018
Detected ib both periods

(A)

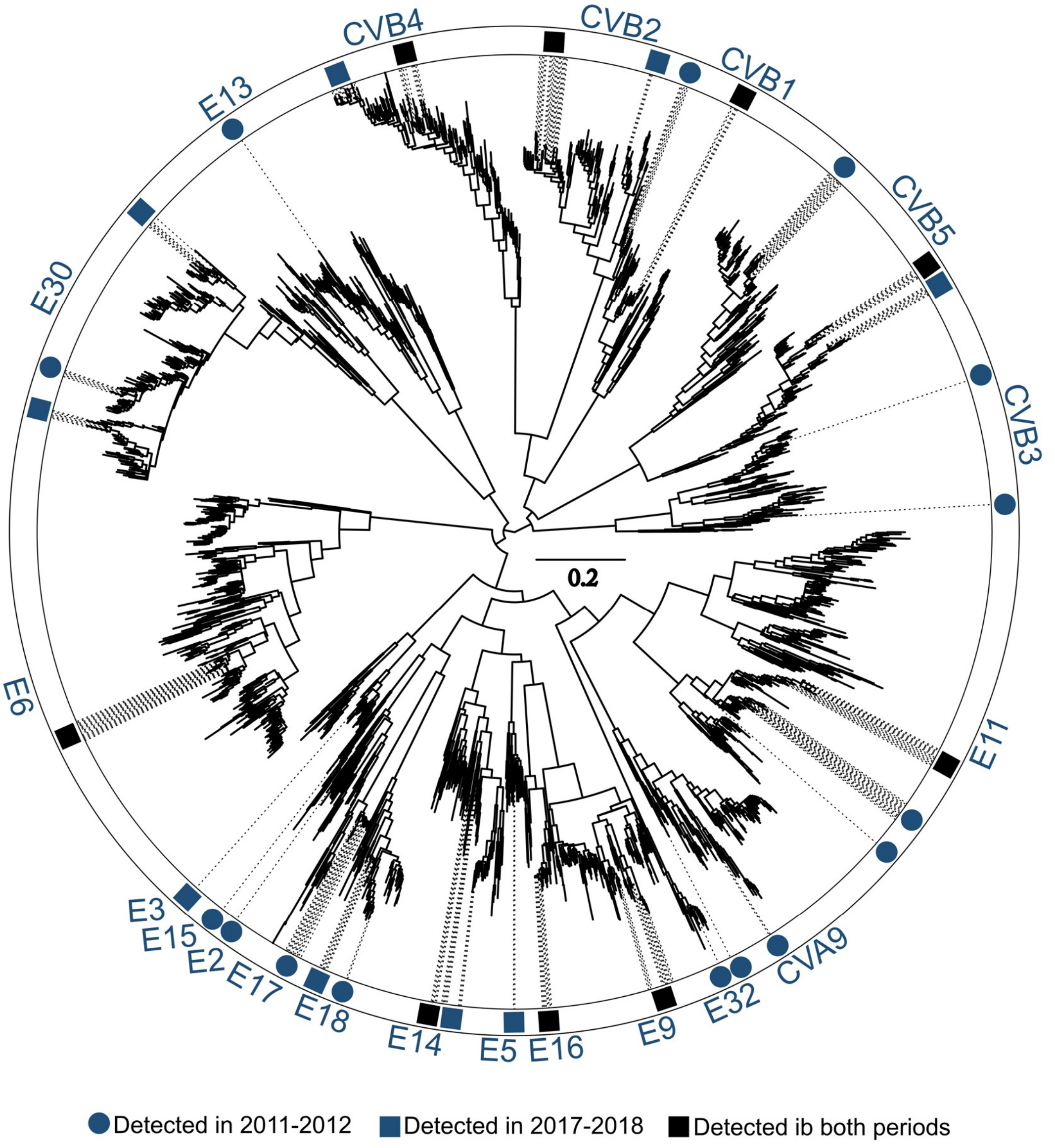
CVB4
CVB2
CVB1
CVB5
CVB3
E11
CVA9
E32
E9
E16
E5
E14
E18
E17
E2
E15
E3
E6
E30
E13
0.2
Detected in 2011-2012
Detected in 2017-2018
Detected ib both periods

(B)

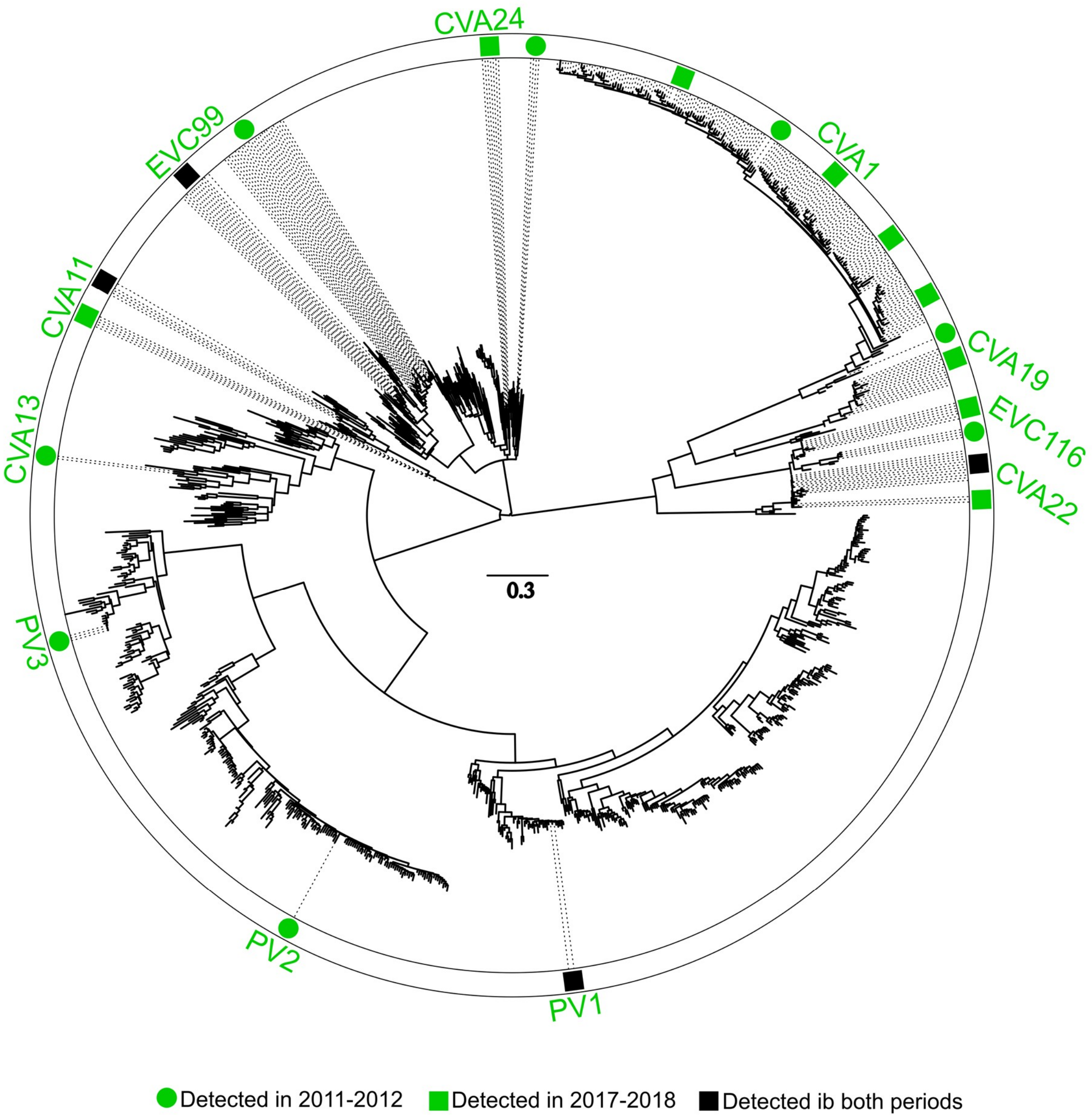

(C)

Figure S1. Maximum likelihood phylogenetic trees of Human Enterovirus species A (**A**), B (**B**), and C (**C**). Each tree was constructed with centroid sequences detected in wastewater samples from Córdoba City (Argentina) during this study and representative sequences of different Human Enteroviruses types reported elsewhere. Dotted lines projecting outward from branches represent centroid sequences. Colored circles and squares indicate the period(s) in which different phylogenetic clusters of this study were detected. aLRT-SH values at type-definition node levels were >0.7 for all types. The bar at the center of each phylogenetic tree denotes genetic distance. CVA: coxsackievirus type A; CVB: coxsackievirus type B; E: echovirus; EVA: enterovirus type A; EVB: enterovirus type B; EVC: enterovirus type C; PV: poliovirus.
